# A highly endemic area of *Echinococcus multilocularis* identified through a comparative re-assessment of prevalence in the red fox (*Vulpes vulpes*), Alto Adige (Italy: 2019–2020)

**DOI:** 10.1371/journal.pone.0268045

**Published:** 2022-05-05

**Authors:** Federica Obber, Roberto Celva, Graziana Da Rold, Karin Trevisiol, Silvia Ravagnan, Patrizia Danesi, Lucia Cenni, Chiara Rossi, Paola Bonato, Katia Capello, Heidi C. Hauffe, Alessandro Massolo, Rudi Cassini, Valentina Benvenuti, Andreas Agreiter, Davide Righetti, Marco Ianniello, Debora Dellamaria, Gioia Capelli, Carlo V. Citterio

**Affiliations:** 1 Istituto Zooprofilattico Sperimentale delle Venezie, Legnaro, Italy; 2 Conservation Genomics Research Unit, Centre for Research and Innovation, Fondazione Edmund Mach, San Michele All’Adige, Italy; 3 Ethology Unit, Department of Biology, University of Pisa, Pisa, Italy; 4 Department of Animal Medicine, Production and Health (MAPS), University of Padua, Legnaro, Italy; 5 Wildlife Management Office, Province of Bolzano, Bolzano, Italy; 6 Ministry of Health, Directorate General for Animal Health and Veterinary Drugs, Rome, Italy; Universitat Autònoma de Barcelona, SPAIN

## Abstract

Surveillance of *Echinococcus multilocularis* at the edge of its range is hindered by fragmented distributional patterns and low prevalence in definitive hosts. Thus, tests with adequate levels of sensitivity are especially important for discriminating between infected and non-infected areas. In this study we reassessed the prevalence of *E*. *multilocularis* at the southern border of its distribution in Province of Bolzano (Alto Adige, northeastern Alps, Italy), to improve surveillance in wildlife and provide more accurate estimates of exposure risk. We compared the diagnostic test currently implemented for surveillance based on coproscopy and multiplex PCR (CMPCR) to a real-time quantitative PCR (qPCR) in 235 fox faeces collected in 2019 and 2020. The performances of the two tests were estimated using a scraping technique (SFCT) applied to the small intestines of a subsample (n = 123) of the same foxes as the reference standard. True prevalence was calculated and the sample size required by each faecal test for the detection of the parasite was then estimated. True prevalence of *E*. *multilocularis* in foxes (14.3%) was markedly higher than reported in the last decade, which was never more than 5% from 2012 to 2018 in the same area. In addition, qPCR showed a much higher sensitivity (83%) compared to CMPCR (21%) and agreement with the reference standard was far higher for qPCR (0.816) than CMPCR (0.298) meaning that for the latter protocol, a smaller sample size would be required to detect the disease. Alto Adige should be considered a highly endemic area. Routine surveillance on definitive hosts at the edges of the *E*. *multilocularis* distribution should be applied to smaller geographic areas, and rapid, sensitive diagnostic tools using directly host faeces, such as qPCR, should be adopted.

## Introduction

*Echinococcus* spp. (Cestoda, Cyclophyllidea, Taeniidae) are small intestinal tapeworms causing zoonoses of public health importance worldwide. In the European Union (EU), it is mandatory to report the detection of these pathogens to national authorities and their surveillance, prevention and control are closely regulated for pets, livestock and wildlife. Collection of relevant metadata is also highly recommended by both the Directive EU 2003/99/EC (monitoring of zoonoses and zoonotic agents) and the Regulation EU 2016/429 (‘Animal Health Law’).

Among these, *Echinococcus multilocularis* has a complex life cycle, depending on a predator-prey system, where the adult (strobilar) stage of the parasite is carried by wild and domestic canids (definitive hosts: DHs), which in turn become infected by ingesting small rodents (intermediate hosts: IHs) carrying the larval stage (metacestode) [1]. *E*. *multilocularis* is the agent of a severe zoonosis, alveolar echinococcosis [2], which affects more than 18,000 new patients/year worldwide [3], 150–200 of which from the endemic area of central-eastern Europe [4].

In 2019, among the 751 human echinococcosis cases reported in the EU, 147 (26.5%) were attributable to *E*. *multilocularis* [5], with an incidence that has been increasing notably in recent decades [4]. In humans, who act as dead-end IHs, transmission is deemed to occur through the faecal-oral route when eggs (oncospheres) are ingested with water, wild berries and mushrooms or garden vegetables contaminated with DH faeces [6], or by hand-mouth contact to eggs derived from the contaminated environment, including skin or coat of definitive hosts [7, 8]. Oncospheres hatch in the gut, penetrate the intestinal wall and by the lympho-hematogenous route reach the liver, where they develop into metacestodes. These multiply asexually, infiltrating the liver and eventually spreading to other organs through a metastasis-like process [9]. Alveolar echinococcosis develops very slowly in humans, taking from a few months [4] to 15 years [10] to become clinically evident, although immune-suppressed patients show faster proliferation with earlier detection [11]. If untreated, prognosis is poor, but even the treatment itself is burdensome, as it includes a combination of surgery and long-term anti-parasitic therapy [11].

In the EU, the DH is predominantly represented by the red fox (*Vulpes vulpes*). From the historically endemic areas of Switzerland, southeast France and southern Germany, *E*. *multilocularis* has been expanding north, to northern France and Scandinavia, as well as east to the Balkans [5, 12–14]. Moreover, in the last two decades this cestode has been found sporadically in southern France [5, 15], as well as in northern Italy in both the Eastern [16] and Western Alps [17], although in the latter area it was found in wolves (*Canis lupus*) and sheep dogs (*C*. *domesticus*; no data are available for foxes). These latter areas currently represent the southernmost border of the parasite’s distribution in the EU. Although high spatial heterogeneity has been noted, in the endemic areas of central and northern Europe the host-parasite-environment pattern seems well established and is expected to be more predictable, whereas the same does not occur at the edges of the distribution [18], where long-term surveillance is crucial to assess possible trends in prevalence and spread, as well as the exposure risk for humans. To this aim, sensitivity of the available diagnostic tests is of paramount importance, since worm burden levels may be low and the presence of *E*. *multilocularis* infections in both DHs and IHs may be very hard to detect.

In the Province of Bolzano (northeastern Alps, Italy), *E*. *multilocularis* was first detected in red foxes in early 2000 at a prevalence of about 13%, estimated using a nested PCR on DNA extracted from fox faeces [16]. In the following years, in order to increase the surveillance area and sample, another test combining coproscopy with multiplex PCR (CMPCR) on parasite eggs was used to analyse 2872 faecal samples across northeastern Italy [19]. While optimizing the cost/benefit ratio, this test was known to have a good specificity (93.4%) but a low sensitivity (54.8%), relying on a preliminary screening by flotation of cestodes’ eggs, so that their burden could drop under the detection threshold [20]. CMPCR was used to confirm the persistence of the Alto Adige focus from 2012 to 2018, with an increasing trend in prevalence in later years, although never higher than 5% annually [19].

Considering the persistence and severity of this zoonosis, the current work aimed to enhance the sensitivity of *E*. *multilocularis* surveillance in red foxes, re-assess its prevalence in the Province of Bolzano, and provide guidelines for a more effective surveillance strategy at the southern edge of its distribution.

## Materials and methods

**Fig 1:**
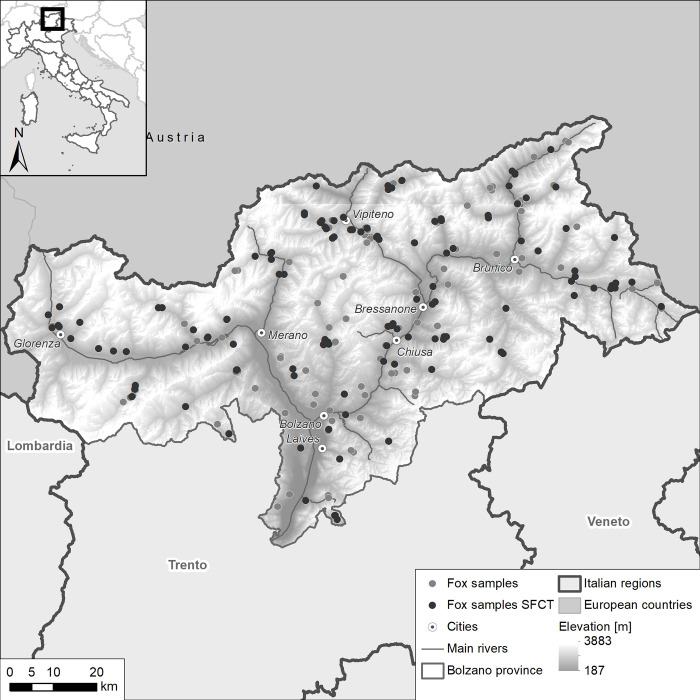
Distribution of individual fox faecal samples from Alto Adige in 2019–2020 (n = 235), indicating the subset examined for adult *Echinococcus multilocularis* worms by SFCT (n = 123). The DEM used as baselayer is provided by TINITALY. The data are available under license CC BY 4.0.

In 2019 and 2020, 235 red foxes were collected across the Province of Bolzano by provincial wildlife technicians and transported to the Bolzano Laboratory of the Istituto Zooprofilattico Sperimentale delle Venezie (IZSVe). Foxes were either legally culled during the hunting season or for local depopulation plans scheduled and approved by national and local wildlife management authorities, performed by gamekeepers and authorized hunters, or found as carcasses during passive surveillance on prioritary diseases under international and national legislation. To achieve an even sampling regime across the territory, one to four animals were collected from each hunting area (Fig 1). At necropsy, a faecal sample was taken from the rectum by a sterile glove. Moreover, when it was intact and not damaged (e.g. by the gun shot), the small intestine was tied off at both ends, removed and stored. Both faecal samples and small intestines were frozen at -80°C for at least 72h to inactivate *Echinococcus* eggs.

Each faecal sample was then thawed and divided into two equal parts and tested for the presence of *E*. *multilocularis* by two methods:

1. CMPCR, following Citterio et al. 2021 [19] (at the Parasitology laboratory of IZSVe in Padova): briefly, 2 g of faecal matter was examined for *Taeniidae* eggs by flotation, filtration and sieving. Multiplex PCR amplification and Sanger sequencing were then performed on harvested eggs.
2. qPCR following Knapp et al. 2016 [21], with minor modifications (at the Animal, Environmental and Antique DNA and Sequencing Platforms of the Fondazione E. Mach): in brief, whole DNA was extracted from 200 mg of faecal sample as described in Santa et al. 2019 [22], using the automated magnetic bead based extraction kit Mag-Bind Stool DNA 96 kit (Omega Bio-Tek, USA) after a freeze-thawing step to facilitate DNA release from eggs. The extraction step was followed by qPCR amplification (Viia 7 Real-time PCR System, ThermoFisher Scientific, Waltham, MA) targeting the mitochondrial DNA marker rrnL, using 10 pmol of species-specific primers for *E*. *multilocularis* (84 bp), as described in Knapp et al. 2014 [23]. To verify the presence of inhibitors in the reaction, 1 μl of an internal control (IAC) plasmid (1000 copies/μl) was added to the solution. This IAC was specifically generated to be amplifiable by the same rrnL gene primers, and kindly provided by the ANSES laboratory in Nancy (National Reference Laboratory for *Echinococcus* spp., Malzeville, France).To detect the fluorescent signal of the IAC plasmid and the parasite DNA amplicon in the reaction, 2 pmol of rrnL hydrolysis rrnL-gene probe and 2 pmol of the IAC-probe were added. The qPCR-positive samples were amplified with the same primer pair and sequenced with the dideoxy chain-termination protocol on an ABI PRISM 3730xl Genetic Analyzer (Applied Biosystems) using the BigDye Terminator cycle sequencing kit (Perkin Elmer, Applied Biosystems Division, Foster City, CA, USA). Sequences were aligned with an *E*. *multilocularis* reference fragment (GenBank acc. n. AB018440) using BioEdit 7.0.9 [24].

In order to evaluate the performances of the two diagnostic tests, a subsample of fox intestines was examined for adult worms by the scraping, filtration and counting technique (SFCT) following Santa et al. 2018 [25]. SFCT is an improvement of the sedimentation and counting technique (SCT), which was previously regarded as the reference standard, although with the addition of a 1 mm sieve filtration step, in Gesy et al. 2013 [26]. For the purposes of this paper, we used SFCT as our reference standard. We performed SFCT on the small intestines of foxes that had tested positive for one or both diagnostic tests in faeces (n = 23), plus 100 faecal-negative samples, for a total of 123. Briefly, the pylorus to caecum section of the small intestine was cut into 30 cm lengths, each of which was opened longitudinally and rinsed with tap water to remove loose faecal content. The intestine was then scraped, the resulting wash water was poured through a series of sieves (1000 μm, 212 μm and 75 μm), and the respective filtrates were collected separately into three distinct beakers. The filtrates were analysed using a stereomicroscope (10X-63X, Nikon SMZ800, Japan) in 5 cm gridded petri dishes, to count the adult helminths. Using the SFCT results, sensitivity (Se) and specificity (Sp) with 95% exact binomial confidence intervals for each test, and their agreement (Cohen’s Kappa) with the reference standard were calculated. True prevalence (TP) was then estimated, using the adjustment to the Rogan-Gladen formula as proposed by Lang and Reiczigel for confidence intervals [27]. For the SFCT dataset, maximum likelihood of the negative binomial exponent k was also estimated by QPweb [28]. Finally, the sample size needed for the detection of *E*. *multilocularis* by each of the two faecal tests was assessed at the estimated TP and according to varying expected prevalence, using the modified binomial approximation analysis method implemented in Epitools (https://epitools.ausvet.com.au/freecalctwo). We set a target population size of 300 individuals on a local scale (hypothetical epidemiological unit), based on the results of unpublished data from wildlife management offices in different areas of the northeastern Italian Alps, providing estimated densities of 2.1, 3.4 and 7.5 foxes/km^2^.

## Results and discussion

All relevant data can be found here and in S1 Table. The distribution of the 235 fox faecal samples and of the 123 fox small intestines in the study area is shown in Fig 1. Of the 235 fox faecal samples, seven (2.9%) were CMPCR-positive and 34 (14.4%) were qPCR-positive. The digital elevation model (DEM) used as a baselayer in the figures is provided by TINITALY [29].

**Fig 2:**
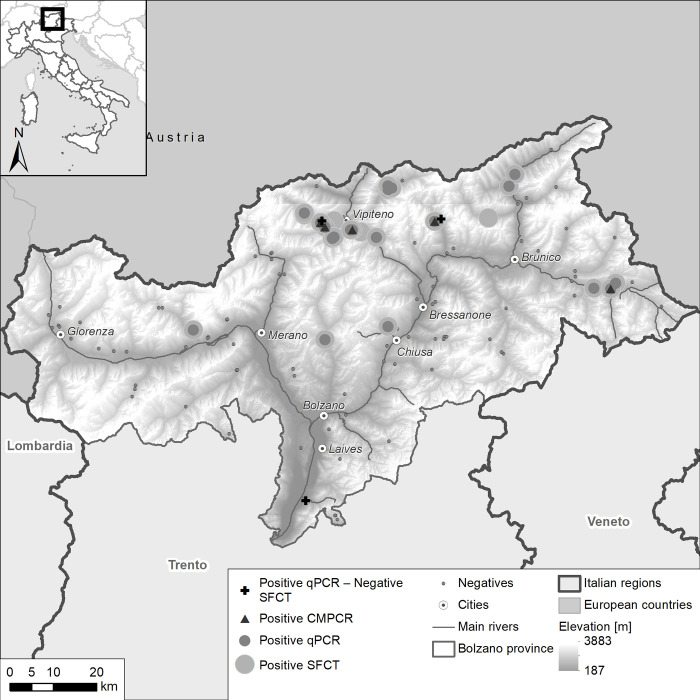
Results of CMPCR, qPCR and the SFCT- reference standard used to detect *Echinococcus multilocularis* in fox faecal samples collected in 2019–2020 (n = 123). The DEM used as baselayer is provided by TINITALY. The data are available under license CC BY 4.0.

Adult *E*. *multilocularis* were found by SFCT in 24/123 (19.5%) fox small intestines (Fig 2) while, for the same animals, five (4.1%) and 23 (18.7%) faecal samples resulted as CMPCR and qPCR positive, respectively. Outcomes for the three diagnostics are summarized in Table 1, whereas *E*. *multilocularis* burden and diagnostic results for SFCT positive samples are presented in S2 Table.

**Table 1:** Matrix of the results of the two faecal tests (CMPCR and qPCR) and the reference standard (SFCT) for the detection of *Echinococcus multilocularis* in 123 foxes.

TEST	CMPCR		qPCR		SFCT	
	POS	NEG	POS	NEG	POS	NEG
CMPCR	**5 (4,1%)**	**118**				
qPCR	5	100	**23 (18,7%)**	**100**		
SFCT	5	99	20	96	**24 (19,5%)**	**99**

The intensity of *E*. *multilocularis* ranged from 4 to 19800 parasites per fox, with a mean abundance of 538.1 parasites/host. The parasite distribution was a negative binomial and highly aggregated (k = 0.022). In most positive animals (16/24), a burden of >50 parasites/host was recorded, and the qPCR was able to detect all these animals, apart from one fox with 164 parasites. On the contrary, the CMPCR missed to detect many animals with high burden of *E*. *multilocularis*, including a fox with 14,276 *E*. *multilocularis*.

Compared to SFCT, CMPCR detected ‘true’ infections in 5/24 (4.1%) cases, resulting in very poor sensitivity (Se) (0.21; Table 2), and far lower than previously reported (about 0.55 [20]). Conversely, qPCR confirmed the SFCT results in 20/24 (86.9%) of the cases, translating into a much higher Se (0.83; Table 2). The qPCR test also identified three positive samples that were negative for SFCT, and failed to detect four positive SFCT samples: these samples were thus considered respectively as false positives and false negatives.

**Table 2:** Sensitivity, specificity and agreement (K) for two diagnostic methods (CMPCR and qPCR) for detecting *Echinococcus multilocularis* in fox fecal samples compared to the SFCT—reference standard.

Index	CMPCR versus SFCT (95% CI)	qPCR versus SFCT (95% CI)
**Sensitivity (Se)**	0.21 (0.07–0.42)	0.83 (0.63–0.95)
**Specificity (Sp)**	1 (0.96–1)	0.97 (0.91–0.99)
**Agreement (K)**	0.30 (0.09–0.50)	0.82 (0.68–0.95)

**Fig 3:**
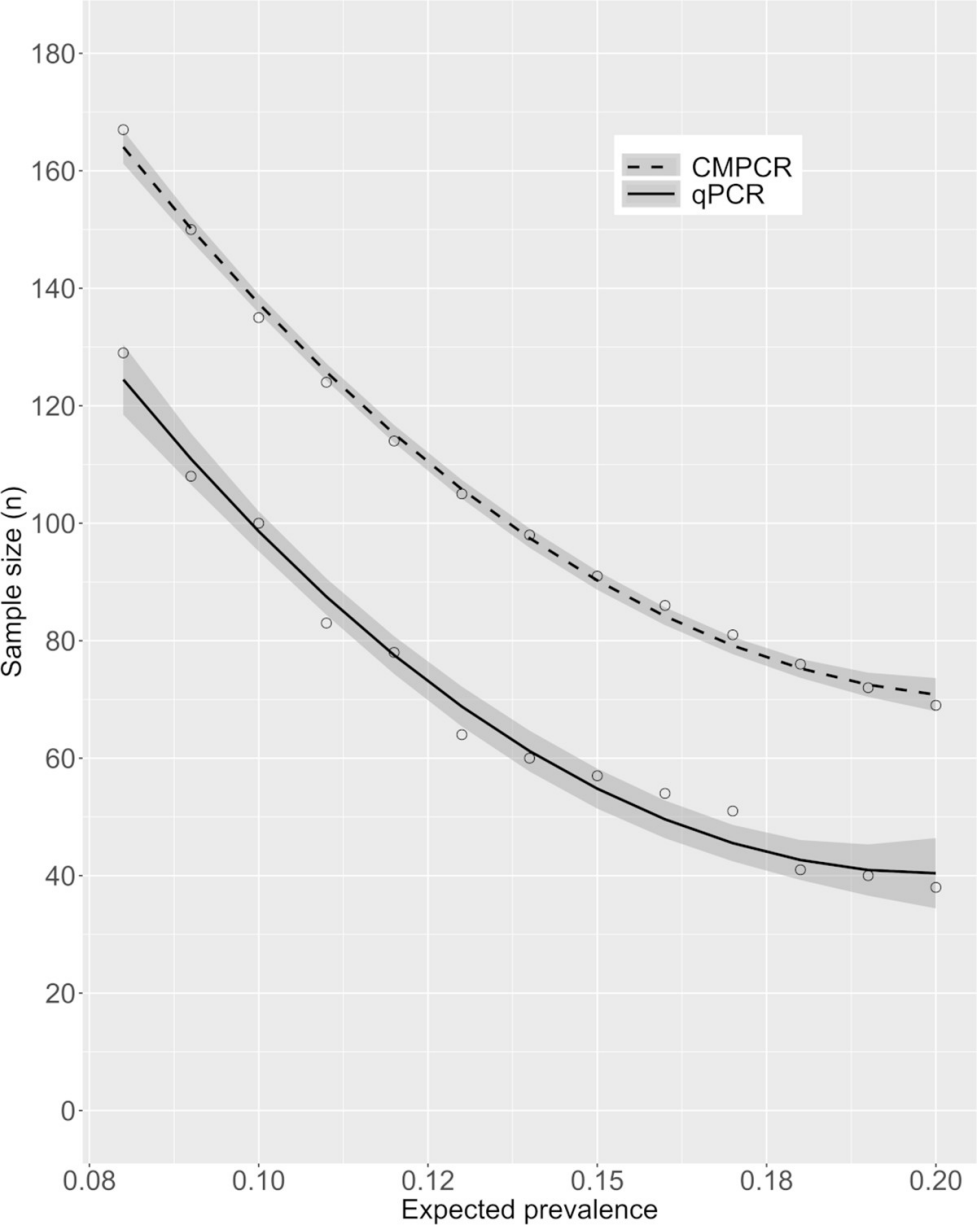
Sample size required to detect *Echinococcus multilocularis* at various expected prevalence estimates (with 95% CI) in a population of 300 foxes.

Based on the Se and Sp calculated from these results (Table 2), the TP estimated for the whole sample set (n = 235) was 14.3% (CI95%: 0–29.14) for CMPCR and 14.2% (CI95%: 7.04–22.64) for qPCR. The sample size required for the detection of *E*. *multilocularis* in a fox population of 300 individuals with various expected prevalence values is shown in Fig 3. Regression was obtained by interpolating 13 prevalence values. For example, if the expected prevalence is 15%, about 90 individuals (about 30% of the population) should be tested when using CMPCR, whereas about 60 individuals (20%) would be enough to detect parasite presence by qPCR.

Our results suggest that the prevalence of *E*. *multilocularis* in Alto Adige was higher than previously reported in the last decade (less than 5%). Indeed, while non-adjusted prevalence of CMPCR (4.1%) is in line with previous studies, the present study provided an estimated true prevalence of about 14% for both the molecular diagnostic tools used for surveillance. As a consequence, according to the standards proposed by Casulli et al. 2015 [30], this region should now be considered as highly endemic (prevalence >10%) [28], suggesting that surveillance should be intensified both locally and in the bordering territories.

The methods most widely used in the EU for detecting *E*. *multilocularis* in animal carcasses are modifications of techniques targeting adult parasites, such as the sedimentation and counting (SCT) and intestinal scraping (IST) [30]; the copro-antigen ELISA test [31]; or PCR [32] for species determination. SCT and IST require animal intestines for screening, and therefore can be used only where hunting/culling and/or passive surveillance plans on foxes are in place, whereas copro-antigens and PCR can be applied on fresh fox scats collected in the field, without the need of necropsy. Moreover, necropsy-based methods aimed at detecting adult helminths have practical limitations, since they require the collection of a consistent number of DHs throughout a study area, storage capacity of large volumes of organs temporarily at -80°C (for ensuring positive samples are not infective) and long-term at -20°C (for storage), as well as being time-consuming (at least 3 hours for each sample). Therefore, indirect diagnostics methods on faeces seem a preferable option for surveillance, given also that faecal samples can be collected in the field using a standardized protocol, and many samples can be screened simultaneously and rapidly. However, a surveillance protocol coupling the monitoring of faeces from both fox carcasses and scats would encompass the life cycle of both the parasite and its main definitive host throughout the year and, additionally, faecal prevalence could be used as a proxy for human risk of exposure. In addition, non-invasive collection and genotyping of scats allow repeated sampling of the same fox social groups, tracking the seasonal pattern of infective eggs shedding by individual foxes. Antigenic and molecular tests are rapid and useful in regions characterized by a high parasitic presence [33], but copro-antigen ELISA has a low sensitivity, especially in areas of low parasitic burden [31]: therefore molecular methods remain the most efficient option. Based on our results, tests directly applied to faeces, such as qPCR, are recommended. In fact, besides reducing the number of samples needed to detect the infection (Fig 3), and consequently the sampling effort and the cost of analyses, qPCR would also be the best option for detecting parasite DNA from scats collected in the field, since these generally result in poor quality DNA compared to fresh faecal samples taken during necropsies [34]. The superior performance of qPCR compared to CMPCR was probably due to the different matrix used for PCR amplification: whereas the former targets DNA obtained by whole-stool extraction, CMPCR follows a delicate pre-extraction phase to minimize the influence of PCR inhibitors (the floatation, filtration and sieving steps). During this procedure, a portion of taeniid eggs is likely to be lost, resulting in highly diluted DNA samples. This could be the cause of the differences in performance between the two molecular techniques. Such performance differences deserve further investigation, which is however beyond the scope of the present paper. The higher sensitivity of qPCR is offset by a somewhat lower specificity, suggested by the three positive samples not identified by SFCT (see also [35]). However, since SFCT is designed to detect adult worms, we cannot exclude that the extra positive samples detected by qPCR were indications of late infections, when traces of parasite DNA may still be present in the intestine but adult helminths are no longer macroscopically detectable; or, on the contrary, of early infections, when immature parasites (scolices) are present but are very difficult to identify microscopically [16]. Since the vast majority of gastrointestinal helminth populations show aggregated distributions, in which most individuals harbour very few or no worms, a sensitive test on faeces becomes even more important. This is further confirmed by our study, in which qPCR showed far better performance compared to CMPCR, particularly in detecting individuals in which *E*. *multilocularis* burden was low.

At the edges of *E*. *multilocularis* distribution, an efficient strategy for surveillance would be to progressively include areas bordering the foci, in which the parasite could have gone undetected, or in which *E*. *multilocularis* had been found occasionally, but no further confirmed, using qPCR as the main diagnostic tool. In such a framework, we suggest that surveillance in foxes should be performed on a small scale (e.g. considering an area similar to an Alpine valley as the basic epidemiological unit) rather than a larger one (e.g. using the NUTS at level 1 or 2, as recommended for *E*. *granulosus* by Tamarozzi et al. 2020 [36]). This would optimize efforts by increasing the chances of detecting *E*. *multilocularis* in areas previously considered free from the parasite. Moreover, such a detailed surveillance would be more informative of the actual risk of exposure for humans.

Finally, it is worth noting that a high endemicity of *E*. *multilocularis*, such as that found in Alto Adige, should be a mandate for increased surveillance of possible human cases, as well as infections in domestic dogs. As dog infection could increase the disease risk to humans, we suggest that this surveillance also use the most sensitive molecular tools available.

## Supporting information

S1 Table Results of CMPCR, qPCR of 235 foxes. *E*. *multilocularis* burden of subset examined for adult worms by SFCT (n = 123).
(XLSX)

S2 Table SFCT positives sample, diagnostic results for CMPCR, qPCR and *E*. *multilocularis* burden. (XLSX)
